# Electrophysiological correlates of spontaneous mind wandering in attention-deficit/hyperactivity disorder

**DOI:** 10.1016/j.bbr.2020.112632

**Published:** 2020-08-05

**Authors:** Natali Bozhilova, Ruth Cooper, Jonna Kuntsi, Philip Asherson, Giorgia Michelini

**Affiliations:** a Social, Genetic and Developmental Psychiatry Centre, Institute of Psychiatry, Psychology and Neuroscience, King’s College London, De Crespigny Park, London, SE5 8AF, United Kingdom; b Newham Centre for Mental Health, Unit for Social and Community Psychiatry, Queen Mary University of London, London, United Kingdom; c Semel Institute for Neuroscience & Human Behavior, University of California Los Angeles, 760 Westwood Plaza, Los Angeles, CA, 9002/4, United States

**Keywords:** ADHD, Mind wandering, Neuroscience, EEG

## Abstract

We recently hypothesised that increased spontaneous mind wandering (MW-S) reflects a core process underlying attention-deficit/hyperactivity disorder (ADHD). Previous studies show that individuals with ADHD and neurotypical individuals with increased MW-S display similar cognitive-performance and electrophysiological (EEG) impairments in attentional processes. However, the cognitive-EEG markers associated with increased MW-S in ADHD remain poorly understood. We therefore investigated such markers in a sample of 69 sex- and age-matched adults with ADHD and 29 controls during the Sustained Attention to Response Task. We compared task performance and EEG measures (P3, time-frequency brain-oscillations) of attentional processes between groups, and examined their association with a validated self-report questionnaire of MW-S. Finally, we tested the hypothesis that MW-S and ADHD diagnosis relate to the same cognitive-EEG impairments using a hierarchical regression model. Compared to controls, adults with ADHD showed attenuations in P3, event-related alpha and beta suppression during response inhibition (No-Go trials), and theta power activations during response execution (Go trials), as well as increased reaction time variability and more commission/omission errors. MW-S was also continuously associated with most cognitive-EEG measures related to ADHD. The hierarchical regressions on measures associated with both ADHD diagnosis and MW-S showed that MW-S did not explain additional variance in the cognitive-EEG markers (except for beta suppression) beyond ADHD diagnosis, and vice versa. These findings are consistent with our hypothesis that ADHD diagnosis and MW-S share common neural deficits, and that MW-S may reflect a core symptom of the disorder.

## Highlights

• Spontaneous mind wandering (MW-S) is often reported as excessive and highly debilitating by adults with ADHD.
• ADHD and MW-S share common cognitive and neural deficits, which may represent common neurobiological mechanisms of attentional impairment.
• MW-S may reflect a core symptom of ADHD.

## 1 Introduction

Attention-Deficit/Hyperactivity Disorder (ADHD) is a common neurodevelopmental disorder affecting 5–6% of children [1] and 3–4% of adults worldwide [2]. Diagnostic criteria for ADHD focus on developmentally inappropriate and impairing levels of inattentive, hyperactive and impulsive behaviours. These criteria reflect the behavioural symptoms commonly reported by parents and teachers about children with this condition. However, based on the subjective experiences of individuals with ADHD, we recently highlighted the potential role of excessive spontaneous mind wandering (MW-S) on ADHD-related impairments in daily life [3,4]. Based on a narrative review of the literature on MW-S and ADHD, we proposed that spontaneous, uncontrolled and task-irrelevant thoughts, as opposed to controlled, goal-oriented, deliberate mind wandering (MW-D), might provide a potential mechanism underlying cognitive, behavioural and functional impairments in individuals with ADHD [5]. This MW perspective hypothesises that MW-S in individuals with ADHD will have the same neural correlates as ADHD itself [5]. Yet, this hypothesis remains to be formally tested in ADHD samples.

Available studies investigating the neural correlates of MW have mainly used functional magnetic resonance imaging (fMRI) [[6], [7], [8], [9]]. However, understanding the stages of neural processing relevant to fast-changing and covert cognitive processes such as MW may be better investigated with the millisecond temporal resolution of electroencephalography (EEG) [5,10]. EEG studies of MW have included thought probes to detect periods of task-unrelated and task-focused thoughts and mostly focused on P3 event-related potentials (ERP) (although also see findings by Braboszcz and Delorme [28]; Kam et al. [11] on the N1). In this context, thought probes are experience sampling questions during tasks that require sustained attention, which enquire about whether the person is focused on the task or not. These studies have shown reduced P3 during periods of MW compared to periods of focused attention on the task [[12], [13], [14], [15]], which may reflect deficits in attention allocation, as well as poorer response inhibition [16].

Reduced P3 has also been consistently found in individuals with ADHD during attentional tasks [17,18]. Furthermore, using source localisation, previous studies in individuals with ADHD have found that alterations in the large-scale networks implicated in MW (e.g. fronto-parietal network [FPN], default mode network [DMN] and ventro-attentional network [VAN]) are associated with reduced P3 amplitude [19,20]; as well as with increased reaction time variability (RTV) [21], a key cognitive impairment associated with ADHD and thought to reflect lapses in attention [22]. Based on a recent meta-analysis [23], a moderate attenuation in P3 has been reliably identified in across dozens of studies. Reduced P3 has also often been reported during MW episodes during sustained attention tasks (e.g., SART) [13,14,24]. However, no study has investigated whether reduced P3 is associated with MW-S in individuals with ADHD.

Beside investigations of ERPs, other studies have examined EEG spectral power during MW episodes in population-based samples. Using thought probes and quantitative EEG (qEEG) analyses, parietal alpha power increased during episodes of MW during vigilance [25], Stroop [26] and switching [27] tasks. Braboszcz and Delorme [28] investigated quantitative EEG power in a population-based sample. Instead of using thought probes, in another qEEG study, this study required participants were required to press a button as soon as they noticed task-unrelated thoughts during a breath focus task and compared these periods of MW with periods of deliberate attention on the breath [28]. Compared to periods of breath focus, self-caught MW was characterised by greater theta power and lower alpha and beta power in the earlier window (the first 300−400 ms after reporting MW), which the authors interpreted as reflecting decreased alertness and early perceptual processing during MW [28]. Two further studies, using finer grained time-frequency brain oscillatory analyses, similarly found reduced event-related theta and beta during early stimulus processing (250−500 ms) after reporting either self-caught [29] or probe-caught MW [30]. In the latter study, both frontal and parietal alpha and centro-parietal beta were reduced during MW compared to task focus in a later time window (500−750 ms) [30]. Another study using finer grained time-frequency brain-oscillatory analyses similarly found increased event-related theta power and decreased beta power during periods of self-caught MW compared to on-task periods [29]. These EEG markers may therefore reflect neural correlates of self-caught MW.

Alterations in brain oscillations have also been reported in ADHD samples. Using q EEG analyses, posterior alpha power, thought to reflect attentional selection and activation (also commonly referred to as attention inhibition/gating [31]), was increased in individuals with ADHD compared to controls during a sustained attention task [32], and showed a familial association with the disorder [33]. More detailed time-frequency analyses further reported attenuated event-related alpha suppression in individuals with ADHD, show attenuated event-related alpha suppression, under high cognitive demands [34] and during attentional performance [[35], [36], [37]]. Attenuated alpha suppression during visual attention tasks has also been linked to task performance, including increased RTV and omission errors, in individuals with ADHD [38], as well as failure to suppress activity in task-irrelevant (sensorimotor) regions [39]. Further initial evidence in individuals with ADHD indicates reduced event-related theta power (reflecting reduced attention allocation) and increased theta phase variability (neural variability in stimulus processing over trials) during an attentional task [37,40], which were also associated with greater RTV [40]. Reduced event-related beta suppression, thought to be a marker of motor response activity, has also been reported in adults with ADHD during an attentional task [35]. Overall, these findings suggest that reduced alpha and beta suppression, as well as reduced evoked theta power and increased theta phase variability, may be closely linked to attentional impairments in individuals with ADHD, which may manifest in difficulty inhibiting task-irrelevant and spontaneous thoughts. As such, these markers might also be related with MW-S, but to the best of our knowledge no study to date has examined the association between these EEG impairments and MW-S in ADHD.

The present study investigates whether cognitive-EEG measures relevant to attentional processes during a task probing inhibitory control and sustained attention with Go and No-Go conditions (Sustained Attention to Response Task; SART) are impaired in adults with ADHD, and are significantly associated with severity of MW-S. Firstly, we compare adults with and without ADHD on ERP and finer-grained time-frequency indices of brain oscillations that were sensitive to ADHD-control differences or associated with MW in population samples in the aforementioned studies (Aim 1). Based on this previous literature, we predicted that the ADHD group would show poorer task performance, reduced P3, event-related theta power, alpha and beta power suppressions, and increased theta phase variability. Secondly, we examine the association between these cognitive-EEG measures and self-reported MW-S (using a self-reported questionnaire of MW-S in ADHD) (Aim 2), predicting that MW-S would be continuously associated with the same cognitive-EEG measures sensitive to ADHD-control differences. Finally, to formally test the hypothesis that the same neural deficits underlie increased MW-S and the ADHD diagnosis [5], we analyse MW-S and ADHD jointly into a hierarchical regression model to examine whether impairments in the cognitive-EEG measures were explained by shared or specific effects of ADHD diagnosis and MW-S (Aim 3).

## 2 Method

### 2.1 Participants

The total sample consisted of 69 adults with ADHD and 29 controls. These were selected from a study of 111 adult participants (81 adults with ADHD and 30 control adults) who took part in a randomised placebo-controlled trial of a fatty acid supplements [41]. The remaining participants (12 adults with ADHD and 1 healthy control) were excluded from the analyses due to technical issues (see EEG recording and analyses). Pre-randomisation baseline data were used for the present study. The two groups were matched on age, sex and IQ (Table 1).

**Table 1:** Descriptive statistics and group comparison on demographic information.

	ADHD	Controls		
	Mean ± SD	Mean ± SD	*d*	*p*
Age (years)	33.5 ± 10.26	29.51 ± 8.80	0.42	0.06
IQ	110.16 ± 13.15	111.32 ± 11.74	0.65	0.41
	Males:Females	Males:Females	Chi^2^	p
Gender	44:37	16:14	0.01	0.84

Abbreviations: ADHD – Attention-deficit/hyperactivity disorder, IQ – Wechsler Abbreviated Scale of Intelligence, WASI-II.

Individuals with ADHD were recruited from South London and Maudsley NHS Trust ADHD clinics, online advertisements via adult ADHD networks and primary care physicians. Controls were recruited via recruitment advertisements in the local community. Participants in both groups were excluded if they had a current or past diagnosis of major neurological disorders (e.g. neurological disease, head injury), severe recurrent mental health problems other than ADHD (e.g. psychosis, major depression, bipolar disorder), current or past substance abuse (defined as more than 8 units for males or 6 units for females of alcohol consumed daily, or recreational drug use more than twice weekly), or an IQ < 80.

All the ADHD participants met DSM-5 criteria for ADHD. Participants in the ADHD group were either on stable treatment with ADHD medication (stimulants, N = 48 or atomoxetine, N = 3) or no medication (N = 18). Some ADHD participants (N = 15) were taking a low dose of concomitant medication for depression or anxiety disorders. All control participants screened below threshold for ADHD on the Adult Self Rating Scale for ADHD [42] and were not being treated for any mental health condition.

### 2.2 Procedure

All participants underwent an in-person assessment lasting around 4 h 30 min, which involved a diagnostic interview, a cognitive-EEG assessment, IQ testing (vocabulary and matrix reasoning from the Weschler Abbreviated Scale of Intelligence – II [WASI-II]) and ADHD-related self-report questionnaires. Participants on medications for ADHD were asked to stop taking their medication for 48 h before the research assessment. All participants were asked to refrain from drinking caffeine or smoking on the day of assessments and the preceding evening.

### 2.3 Measures

#### 2.3.1 Spontaneous mind wandering (MW-S): MW hypothesis

MW-S was measured using the Mind wandering Excessively Scale (MEWS), a 12-item self-report measure reflecting descriptions of MW in ADHD. We previously found that the MEWS was highly correlated with the spontaneous mind wandering scale [43] used by Seli and colleagues, who first identified the association between ADHD and MW-S [44]. As such, we used the MEWS to operationalise MW-S. The 12-item Mind Excessively Wandering Scale (MEWS) captures the subjective experience of MW typical of individuals with ADHD, including thoughts constantly on the go, thoughts flitting from one topic to another, and multiple thoughts at the same time [3].

The scale shows excellent internal consistency (α > .90), as well as high sensitivity and specificity (both around 90%; AUC = .97; [3]) to discriminate individuals with and without ADHD, and has been validated as a measure of MW-S in two clinical samples (including the current study sample) [3] and in a large population sample [4]. This scale was shown to be unidimensional with the same factor structure indicating that the same construct/process is being measured across ADHD, controls, male and female groups [4]. Regarding validity, the MEWS shows strong positive correlations with measures of ADHD symptoms and functional impairment in daily life. In the current sample, the MEWS was significantly correlated with inattention (*r* = .77), hyperactivity/impulsivity (*r* = .69), and ADHD-related functional impairment (*r* = .81) [3]. The scale correlated highly with a previously used measure of MW-S [43] (*r* = 0.76, *p* < 0.001), but not MW-D (*r* = 0.05, *p* = 0.06), indicating that MW-S (and not MW-D) is captured by the MEWS in both individuals with ADHD and controls.

#### 2.3.2 Sustained attention to response task (SART)

The SART is a computerised go/no go task measuring both response inhibition and sustained attention. It consists of nine digits presented in random order on a computer monitor. Participants are instructed to withhold responses to the digit 3 (No-Go trial, 11%) but to respond with a button press after all other digits (Go trial, 89%). Participants completed the SART over three blocks, each lasting approximately 5 min. Individual blocks consisted of 225 digits, with each digit presented 25 times and an inter-stimulus interval of 1000 ms.

The following performance indices were measured: commission errors (CE; responses to No-Go stimuli) omission errors (OE; non-responses to Go stimuli), mean reaction time (MRT) and reaction time variability (RTV; measured as standard deviation of reaction times). MRT and RTV were computed on trials with correct responses to Go stimuli only.

### 2.4 EEG recoding and analyses

The EEG was recorded from a 62-channel DC-coupled recording system (extended 10–20 montage), using a 500 Hz sampling rate, impedances under 10 kΩ, and FCz as the recording reference. The electro-oculograms were recorded from electrodes above and below the left eye and at the outer canthi. The EEG data were analysed using EEGLAB [45]. The raw EEG data were down-sampled to 256 Hz, re-referenced to the average of all electrodes, and digitally filtered using basic Finite impulse response (FIR) filters below 1 Hz and above 30 Hz. Prior to re-referencing, flat channels, or channels with extremely large artefacts were interpolated. Sections of data >200 μV were automatically rejected.

Ocular artefacts (blinks and lateral eye movements), clearly isolated heartbeat, line noise and muscle artefacts were identified using independent component analysis (ICA) with the AMICA (Adaptive Mixture ICA) algorithm [46]. ICA allows for the correction of artefactual data through removal of the artefactual components and back-projection of all but those components. Following the back-projection, all datasets were also visually inspected and sections of data containing residual artefacts were removed manually.

Only participants with at least 20 artefact-free EEG segments in each condition were included in ERP/EEG analyses, since at least 20 artefact-free EEG segments are required to observe reliable neural effects and obtain valid ERP/ERSP indices [65]. From the original sample of 111 participants, 4 individuals with ADHD were excluded because of incomplete EEG recordings and an additional 8 were excluded because they had less than 20 artefact-free No-Go trials. One control was also excluded due to poor data quality (extremely large, movement-related artefacts). This left a final sample of 69 individuals with ADHD and 29 controls. Formal power calculations indicate 80% power to detect medium effects sizes (d > 0.50) as statistically significant (α = 0.05) with the current sample (n = 98).

**Fig. 1:**
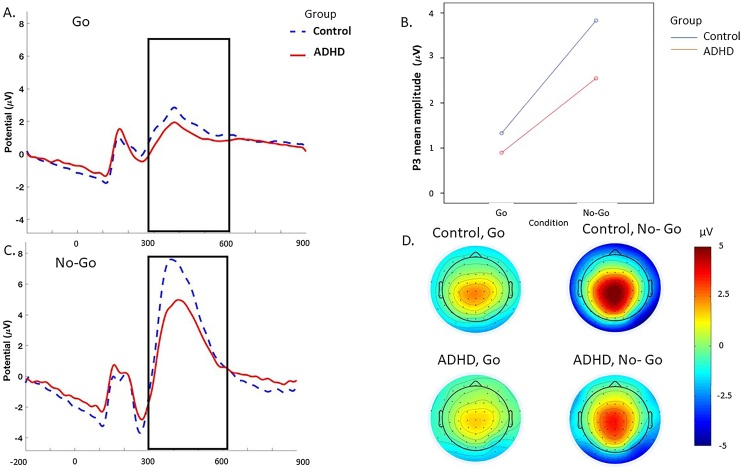
Grand average stimulus-locked event-related potentials of the P3 at the Cz electrode between 300 and 600 ms in ADHD group (red) and control group (blue) across the No-Go and Go conditions: **A.** Grand average during the Go. B. Condition by group interaction. **C.** Grand average during the No-Go stimulus. D. Topographic maps. (For interpretation of the references to colour in this figure legend, the reader is referred to the web version of this article.)

For ERP analyses, stimulus-locked epochs (stimulus window from −500 to 1000 ms) were averaged based on Go trials with correct responses and No-Go trials with no responses (i.e., correctly inhibited responses). Baseline correction was performed using a 500-ms pre-stimulus period. This window was chosen for consistency with the pre-stimulus used for ERSP analyses, where a −500 pre-stimulus baseline correction was chosen for normalisation of ERSPs to capture two full cycles at the lowest frequency of interest (4 Hz theta). ERP measures were identified within the selected electrodes and latency windows for which effects were expected to be largest, based on previous ADHD or MW studies [14,37,47] and verified against the topographic maps and the grand averages (Fig. 1). ERPs were quantified as mean amplitudes within selected windows, which eliminates the effect of peak latency variability [48]. The P3 was measured at Cz between 300 and 600 ms in Go and NoGo trials.

**Fig. 2:**
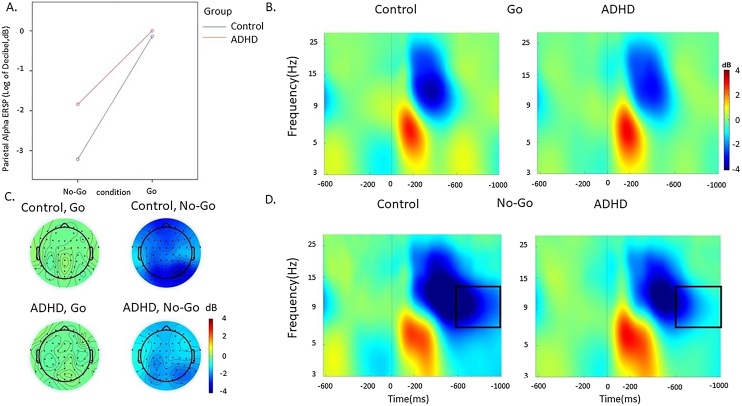
Alpha event-related spectral perturbation (ERSP) at parietal regions in the ADHD and control groups in the Go and No-Go condition during the SART. A. Condition effects in the 600-1000 ms window by group (ADHD group in red, control group in blue). B. ERSP in the Go condition. C. topographic maps by group in the 600–1000 ms window at each condition. D. ERSP in the No-Go condition. (For interpretation of the references to colour in this figure legend, the reader is referred to the web version of this article.)

**Fig. 3:**
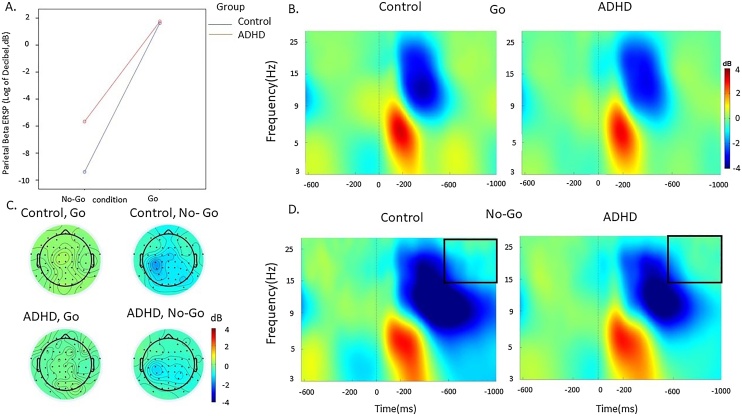
Beta event-related perturbation (ERSP) at parietal regions in the ADHD and control groups in the Go and No-Go condition during the SART. A. Condition effects in the 600-1000 ms window by group (ADHD group in red, control group in blue). B. ERSP in the Go condition. C. topographic maps by group in the 600-1000 ms window at each condition. D. ERSP in the No-Go condition. (For interpretation of the references to colour in this figure legend, the reader is referred to the web version of this article.)

**Fig. 4:**
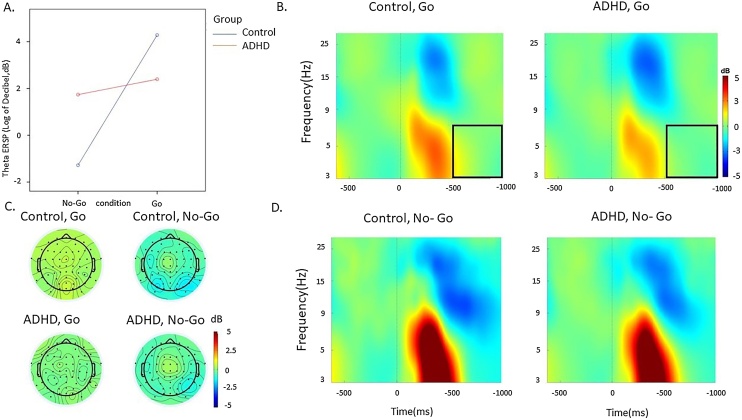
Theta event-related perturbation (ERSP) at fronto-central regions in the ADHD and control groups in the Go and No-Go condition during the SART. A. Condition effects in the 600-1000 ms window by group (ADHD group in red, control group in blue). B. ERSP in the Go condition C. topographic maps by group in the 500-1000 ms window at each condition. D. ERSP in the No-Go condition. (For interpretation of the references to colour in this figure legend, the reader is referred to the web version of this article.)

Time-frequency analyses were used to investigate changes in power and phase related to the Go, correct and incorrect No-Go trials. Power changes were quantified as an event-related spectral perturbation (ERSP) index [45] in a 2500 ms window (from -1000 to 1500 ms) time-locked to the stimuli. The analyses involved Morlet wavelet decomposition of frequencies between 3−30 Hz, with linearly increasing number of cycles (frequency step of 0.80 Hz) from 2 cycles for the lowest frequency (3 Hz) to 24.60 cycles for the highest frequency (30 Hz). This approach optimises the trade-off between temporal resolution at lower frequencies and frequency resolution at higher frequencies, allowing for improved frequency resolution at higher frequencies. This approach was also chosen to measure theta oscillations despite our short task windows and is consistent with several time-frequency studies that also used two cycles at the lowest frequency [30,37,49]. Each ERSP trial was normalised with respect to the mean log-power spectrum from the -500 to 0 ms pre-stimulus period. The ERSP plots display decibel (dB) units of increases (ERS, in red) and decrease (ERD, in blue) in the spectral power at a given frequency and latency with respects to pre-stimulus activity (Fig. 2, Fig. 3, Fig. 4) from which frequency-specific ERSPs can be extracted. Phase consistency was measured as an inter-trial phase coherence (ITC) index calculated from the same Morlet wavelets. The ITC index shows the level of phase consistency of the evoked response across all trials at a given latency and frequency [45,50,51]. ITC values are independent of power and range from 0 (reflecting absence of phase consistency and highest phase variability across trials) to 1 (indicating perfect phase consistency and lowest phase variability).

Stimulus-locked ERSP in the theta (3−7 Hz), alpha (8−13 Hz) and beta (14−30 Hz) bands were extracted in the 1000 ms window. Consistent with previous literature [52,30,53], we segmented the trials into early and late window to measure modulations of power over the trial. In our data, alpha and beta were more prominent after 200 ms, in line with several other studies [27,30,38,49,54]. We therefore measured these rhythms using 200−600 ms and 600−1000 ms for alpha/beta, and 0−500 ms and 500−1000 ms for theta (Fig. 2, Fig. 3, Fig. 4). Reduced phase variability over trials is proposed to underlie stable neural processing of a stimulus, or phasic consistency in the neural response across trials [51]. ITC was measured at stimulus onset in the first window (0−500 ms), where greater phase consistency in response to the event is expected [53]. The ITC analysis was restricted to the theta band, consistent with previous studies reporting a role of this frequency band in phase consistency of the neural response [37,53,55,56] and its association between increased theta phase variability and RTV [40,55]. ERSP and ITC were measured following previous studies and at scalp locations where they were maximal (Fig. 2, Fig. 3, Fig. 4). We identified maximal power changes in the following frequencies: theta over fronto-central areas (average of electrodes: FCz, Cz, C1, C2, FC1, FC2); alpha over parieto-occipital regions (average of electrodes: Oz, O1, O2, P3-P4, P7-P8, POz, PO3-PO4, PO7-PO8) [54,57]; beta over central (average of electrodes: C1-C4, CPz, CP1-CP4) and parietal regions (average of electrodes: PO3-PO7, POz, P3-P8) [54,57].

### 2.5 Statistical analyses

Analysis 1: To test aim 1, differences between individuals with ADHD and controls on performance measures (CE from No-Go trials, OE from Go trials, RTV and MRT from correct Go trials) were investigated using independent sample t-tests. RTV and MRT showed skewed distributions and were log-transformed before analyses, while CE and OE were normally distributed. Group differences on EEG variables, all showing normal distributions, were investigated with general linear models testing main effects of group (ADHD vs control), condition (No-Go vs Go) and two-way group x condition interactions. Since ERSP variables were investigated across two separate windows, three-way group x condition x time window interactions were also investigated. Each frequency band measure was tested in a separate model. When the three-way interaction was not statistically significant, it was dropped from the model and only statistically significant main effects and two-way interactions were included. Significant group and interaction effects were followed up with post-hoc tests.

Analysis 2: To test aim 2, we investigated continuous associations between MW-S (measured with the MEWS) and all the investigated cognitive-EEG measures. In order to report standardised beta coefficients, all measures were first standardised. Cognitive variables were analysed with linear regressions, using MW-S as the independent variable and each of the cognitive variables separately as dependent variables. P3 and ERSP variables measured over different conditions and time windows were investigated with the same general linear models used in analysis 1, but using the MEWS as the independent variable instead of group.

Analysis 3: To address aim 3, we examined the shared and unique variance explained by ADHD and MW-S on the cognitive-EEG measures that showed significant impairments in the ADHD group (in analysis 1) and were also significantly associated with the MEWS (in analysis 2). We ran hierarchical linear regression models with each cognitive-EEG variable (dependent variable); first, entering as independent variables ADHD group in the first block and MEWS in the second block, and, secondly, entering MEWS first and ADHD second.

Given the large number of hypotheses tested in analyses 1 and 2, results were corrected for multiple testing using a false discovery rate (FDR) threshold based on the total number of comparisons. Significant *p*-values for analysis 1 were equal or lower than 0.035 in analysis 1 and equal or lower than 0.010 in analysis 2. Analyses addressing aim 3 were restricted to measures showing ADHD-control differences and significantly associated with the MEWS based on these FDR thresholds, therefore further multiple-testing corrections were not applied.

We have also carried a number of sensitivity analyses: i) comparisons between controls and individuals with ADHD who were not taking medication for anxiety or depression on cognitive-EEG measures, to ensure group differences were not driven by participants taking these medications (Supplementary Analysis 1, Supplementary Table 1); ii) a comparison between individuals with ADHD and controls on ERSP variables over the whole 1 s trial time window (Supplementary Analysis 2); iii) an analysis of theta ITC with theta ERSP (0−500 ms) as a covariate, to account for potential effects of power on the phase of theta [58], (Supplementary Analyses 3); iv) correlations between cognitive performance and EEG variables sensitive to group differences, to examine the behavioural significance of the investigated measures (Supplementary Analysis 4, Supplementary Table 2); v) a re-analysis of the associations of the MEWS with cognitive-EEG variables (Analysis 2) testing whether they differ as a function of group (Supplementary Analysis 5, Supplementary Table 3).

## 3 Results

### 3.1 Analysis 1: differences between ADHD and control groups

#### 3.1.1 Cognitive measures

Compared to controls, individuals with ADHD made significantly more errors (both CE and OE) and showed significantly greater RTV (Table 2). There were no differences between individuals with ADHD and controls on MRT (Table 2).

**Table 2:** Descriptive statistics and group comparison on cognitive-performance and EEG measures.

		ADHD	Control		
		Mean ± SD	Mean ± SD	*d*	*p*
OE		7.06 ± 10.11	2.79 ± 4.64	*0.54*	0.033*
CE		9.76 ± 4.06	7.20 ± 4.20	*0.62*	0.007*
MRT (ms)		328.96±55.88	308.99±43.76	0.39	0.093
RTV (ms)		121.62±59.61	83.37± 26.99	0.83	<0.001**
P3	Go	0.90 ± 0.65	1.33 ± 0.68	*0.65*	<0.001**
	No-Go	2.55 ± 1.29	3.83 ± 1.58	0.89	<0.001**
Alpha ERSP	Go (200−600 ms)	−1.94 ± 1.44	−2.49 ± 1.52	0.37	0.06
	No-Go (200−600 ms)	−2.95 ± 2.27	−3.93 ± 2.28	0.43	0.10
	Go (600−1000 ms)	0.00 ± 0.41	−0.14 ± 0.56	0.27	0.19
	No-Go (600−1000 ms)	−1.84 ± 1.43	−3.21 ± 1.93	*0.77*	<0.0001**
Beta ERSP (Central)	Go (200−600ms)	−1.47 ± 0.72	−1.56 ± 0.72	0.13	0.61
	No-Go (200−600 ms)	−1.86 ± 1.13	−1.94 ± 1.08	0.07	0.75
	Go (600−1000 ms)	0.14 ± 0.220	.16 ± 0.20	0.10	0.68
	No-Go (600−1000 ms)	−0.79 ± 0.85	−1.06 ± 0.75	0.35	0.15
Beta ERSP (Parietal)	Go (200−600 ms)	−1.43 ± 0.77	−1.56 ± 0.70	0.18	0.43
	No-Go (200−600 ms)	−1.95 ± 1.11	−2.38 ± 1.0	0.39	0.04
	Go (600−1000 ms)	0.18 ± 0.220	.16 ± 0.28	0.08	0.81
	No-Go (600−1000 ms)	−0.57 ± 0.68	−0.94 ± 0.80	0.48	0.02*
Theta ERSP	Go (0−500 ms)	1.51 ± 0.84	1.89 ± 0.87	0.44	0.04
	No-Go(0−500 ms)	3.64 ± 1.624	4.17 ± 1.57	0.33	0.14
	Go (500−1000 ms)	0.24 ± 0.220	0.43 ± 0.40	*0.54*	0.005*
	No-Go (500−1000 ms)	0.17 ± 1.10	0.13±1.07	0.12	0.22
Theta ITC	Go (0−500 ms)	0.31 ± 0.07	0.33 ± 0.11	0.24	0.39
	No-Go (0−500 ms)	0.48 ± 0.12	0.50 ± 0.10	0.18	0.35

Abbreviations: ADHD – Attention-Deficit/Hyperactivity Disorder, MRT – MeanReaction Time Variability, CE – commission errors, OE – omission errors, ERSP – Event-related spectral perturbations, ITC – inter-trial phase coherence.

Notes: *significant at p ≤ 0.035 FDR correction, **significant at p ≤ 0.001, Bold: d ≥ .80 indicating large effect size, Italics: d ≥ .50 indicating a medium effect size, d ≥ .20 indicating a small effect size.

#### 3.1.2 ERP and time-frequency measures

*P3.* A significant main effect of condition (*p* < 0.0001), group (*p* < 0.0001) and condition-by-group interaction (*p* = 0.001) was observed for the P3. Post-hoc analyses found that individuals with ADHD had a lower P3 amplitude in both Go and No-Go conditions compared to controls (Table 2), with differences between groups being smaller in the Go compared to the No-Go condition, as indicated by the aforementioned significant interaction. P3 amplitude was larger in the No-Go compared to the Go condition in both groups (*p* = 0.001).

*Alpha ERSP.* A significant main effect of condition (*p* < 0.001), group (*p* = 0.008) and a group-by-condition-by-time interaction (*p* = 0.003) emerged for alpha ERSP. In the 200−600 ms post-stimulus window, there was a main effect of condition (*p* < 0.001), but no significant group-by-condition interaction (*p* = 0.13), and main group effect did not reach statistical significance or survive correction for multiple comparisons (*p* = 0.06). Both groups had lower alpha ERSP (i.e. greater suppression) in the No-Go compared to the Go condition in the 200−600 ms window (*p* < 0.001). In the 600–1000 ms window, there were significant effects of condition (*p* < 0.001), group (*p* < 0.001) and condition-by-group interaction (*p* < 0.001). Alpha ERSP was higher (i.e. suppression was lower) in the ADHD group compared to the control group in the No-Go condition, but not in the Go condition (Table 2).

*Beta ERSP.* A significant main effect of condition (*p* < 0.001), but no effects for group (*p* = 0.63) or group-by-condition-by-time (*p* = 0.49) emerged for beta ERSP over central regions. Both in the 200−600 ms and in the 600−1000 ms window there was a main effect of condition (both *p* < 0.001), but no significant effect of group (*p* = 0.99 and *p* = 0.20 respectively) or condition-by-group interaction (*p* = 0.29 and *p* = 0.14, respectively). Both groups had lower central beta ERSP in the No-Go compared to the Go condition in both time windows (*p* < 0.001).

For beta ERSP over parietal regions, there was a significant main effect of condition (*p* < 0.001), but no significant group-by-condition-by-time interaction (*p* = 0.54), and the main group effect did not reach statistical significance or survive correction for multiple comparisons (*p* = 0.06). In the 200−600 ms window there was a significant main effect of condition (*p* < 0.001) and a significant group-by-condition interaction (*p* = 0.03), but no significant effect of group (*p* = 0.17). The ADHD group showed higher beta ERSP (i.e. lower suppression) in the in the No-Go condition compared to the control group, although this difference did not survive correction for multiple comparisons; no group differences emerged in the Go condition (Table 2). In the 600−1000 ms, a significant main effect of condition (*p* < 0.001), group (*p* = 0.03) and group-by-condition interaction (*p* = 0.03) emerged. The ADHD group showed greater parietal beta ERSP (lower suppression) in the 600−1000 ms compared to the control group in the No-Go condition, while the groups did not differ in the Go condition (Table 2).

*Theta ERSP.* A significant main effect of condition (*p* < 0.001) and a group-by-condition-by-time interaction (*p* = 0.01), but no main effect of group (*p* = 0.23), emerged for theta ERSP. In the 0–500 ms time window, there was a significant effect of condition (*p* < 0.001), but no statistically significant main effect of group (*p* = 0.08) or condition-by-group interaction (*p* = 0.60). Theta ERSP was higher during the No-Go compared to the Go condition (*p* < 0.001). In the 500−1000 ms time window, there was a significant group-by-condition interaction (*p* = 0.035), but no main effect of group (*p* = 0.68), and the main group effect reached statistical significance (*p* = 0.048), but did not survive correction for multiple comparisons. Post-hoc analyses revealed that the ADHD group had lower theta ERSP in the Go condition in the 500−1000 ms compared to controls, but there was no difference in the No-Go condition (Table 2).

*Theta ITC.* A significant main effect of condition (*p* < 0.001), but no main effect of group (*p* = 0.95) emerged for theta ITC. The group-by-condition interaction (*p* = 0.04) reached statistical significance, but did not survive correction for multiple comparisons. Phase consistency was greater in the No-Go compared to the Go condition in both groups (*p* < 0.001).

Sensitivity analyses removing ADHD participants who were taking medication for anxiety or depression yielded results comparable to those of Analysis 1 in the full sample (Supplementary Analysis 1, Supplementary Table 1). Analyses of ERSP measures analysed over the 1 s window showed that the differences between individuals with ADHD and controls in alpha and beta in the No-Go condition remained (Supplementary Analysis 2). However, the differences between groups in theta in the Go condition was no longer statistically significant (Supplementary Analysis 2), indicating that group differences in this measure might be specific to 500−1000 ms window, rather than distributed over the whole 1 s window. Analyses on the potential effect of power on ITC [58], showed that results were unchanged when we repeated analyses of theta ITC controlling for theta power in the same time window (Supplementary Analysis 3). Finally, all the EEG measures sensitive to group differences were correlated to cognitive performance (Supplementary Analysis 4, Supplementary Table 2), indicating that these neural markers are behaviourally significant.

### 3.2 Analysis 2: association between MW-S and cognitive-EEG measures

MW-S was not associated with CE, and the association with OE did not survive FDR correction for multiple comparisons. Instead, the associations with MRT and RTV were positive and survived the FDR threshold. MW-S also showed positive significant associations with alpha and parietal beta ERSPs in the 600−1000 ms window during No-Go trials; and negative significant associations with No-Go and Go P3 amplitudes, Go theta in the 0−500 ms and 500−1000 ms windows, and No-Go theta in the 0−500 ms window (Table 3). These associations also did not differ as a function of group (Supplementary Analysis 5, Supplementary Table 3).

**Table 3:** Linear association between the MEWS and neurocognitive measures in the entire sample.

Association with MW				
		β	95% CIs	*p*
OE		.23	.03;.44	.028
CE		.08	−.002;.38	.053
MRT (ms)		.27	.07;.47	.010*
RTV (ms)		.25	.06;.45	.010*
P3	No-Go	−.39	−.58;−.20	<0.0001**
	Go	−.32	−.46;−.07	.002*
Alpha ERSP	Go (200−600 ms)	.05	−.16:26	.62
	No-Go (200−600 ms)	.09	−.12;29	.40
	Go (600−1000 ms)	.11	−.10:32	.29
	No-Go (600−1000 ms)	.35	.15;.54	.001*
Beta ERSP (Central)	Go (200−600 ms)	−.012	−.22;.19	.91
	No-Go (200−600 ms)	.083	−.12;.29	.43
	Go (600−1000 ms)	−.11	−.31;.10	.31
	No-Go (600−1000 ms)	.23	.03;.43	.023
Beta ERSP (Parietal)	Go (200−600 ms)	.046	−.16;.25	.66
	No-Go (200−600 ms)	.13	−.08;.34	.21
	Go (600−1000 ms)	.012	−.19;.22	.91
	No-Go (600−1000 ms)	.32	.12;.51	.002*
Theta ERSP	Go (0−500 ms)	−.34	−.53;-.15	.001*
	No-Go (0−500 ms)	−.28	−.48;-.09	.005*
	Go (500−1000 ms)	−.26	−.46; −.06	.010*
	No-Go (500−1000 ms)	−.09	−.30; .12	.39
Theta ITC	Go (0−500 ms)	−.10	−.31;.10	.33
	No-Go (0−500 ms)	−.22	−.42;-.02	.035

Abbreviations: CE – commission errors, OE – omission errors, RTV – reaction time variability MRT – mean reaction time.

Notes: *significant at *p* ≤ .01 FDR correction, **significant at *p* ≤ .001.

### 3.3 Analysis 3: association of ADHD and MW-S with cognitive-EEG impairments

Given the high association between ADHD and MW-S in this sample [3], we ran hierarchical regressions including both ADHD status and MW-S to test whether cognitive-EEG impairments were explained by shared or unique effects of these variables. Six cognitive-EEG measures that were both sensitive to ADHD-control differences and significantly associated with the MEWS were carried forward in hierarchical regressions: RTV, Go and No-Go P3, No-Go alpha ERSP between 600−1000 ms, parietal No-Go beta ERSP between 600−1000 ms and Go theta ERSP between 500−1000 ms.

*RTV.* ADHD status entered into block 1 explained 33% of the variance in RTV, F (189) = 10.87, *p* = 0.001. The MEWS score added in block 2 did not significantly increase the variance explained (*R*^2^ change = .002, F change (189) = 0.16 *p* = 0.69). Similarly, the MEWS entered into block 1 explained 27% of the variance, F (190) = 6.84, *p* = 0.01. ADHD status entered in block 2 did not produce a significant increase in variance explained (*R*^2^ change = 0.039, F change (189) = 3.86, *p* = 0.053).

*Go P3*. ADHD status was entered into block 1 explained 9% of the variance in the Go P3, F (189) = 8.68, *p* = 0.004. The MEWS score entered in block 2 did not significantly increase the variance explained (*R*^2^ change = 0.023, F change (189) = 2.29, *p* = 0.13). Similarly, the MEWS entered into block 1 explained 10% of the variance, F(1,90) = 10.23, *p* < 0.02. ADHD status entered in block 2 did not produce a significant increase in variance explained (*R*^2^ change = .009, F change (189) = 0.88, *p* = 0.35).

*No-Go P3.* ADHD status entered in block 1 explained 15% of the variance in the No-Go P3 (F (189) = 6.59, *p* = 0.012). The MEWS score entered in block 2 did not significantly increase the variance explained (*R*^2^ change = .017, F change (189) = 1.82, *p* = 0.18). Similarly, the MEWS entered in block 1 explained 16% of the variance (F(1,90) = 16.61, *p* < 0.0001., ADHD status entered in block 2 did not produce a significant increase in variance explained (*R*^2^ change = .027, F change (189) = 2.91, *p* = 0.09).

*Alpha ERSP*. ADHD status entered into block 1 explained 12% of the variance in No-Go Alpha ERSP (600–1000 ms), F (189) = 12.02, *p* = 0.001. The MEWS total score entered in block 2 did not significantly increase the variance explained (*R*^2^ change = .014, F change (189) = 1.43, *p* = 0.24). Similarly, the MEWS entered in block 1 explained 12% of the variance, F (189) = 12.60, *p* = 0.001. ADHD status in block 2 did not produce a significant increase in variance explained (*R*^2^ change = .030, F change (189) = 3.09, *p* = 0.08).

*Beta ERSP*. ADHD status entered in block 1 explained 6% of the variance in parietal No-Go Beta ERSP (600–1000 ms), F (189) = 5.30, *p* = 0.024. The MEWS total score entered in block 2 produced a significant increase of 5% in variance explained (*R*^2^ change = 0.05, F change (189) = 4.60, *p* = 0.035). The MEWS entered in block 1 explained 10% of the variance, F (189) = 10.22, *p* = 0.002. ADHD group in block 2 did not produce a significant increase in variance explained (*R*^2^ change = .00003, F change (189) = 0.003, *p* = 0.10).

*Theta ERSP*. ADHD group status entered in block 1 explained 7% of the variance in Go Theta ERSP (500–1000 ms), F (189) = 6.59, *p* = 0.012. The MEWS total score in block 2 did not significantly increase the variance explained (*R*^2^ change = .008, F change (189) = 0.78, *p* = 0.38). Similarly, the MEWS entered in block 1 explained 7% of the variance, F (189) = 6.89, *p* = 0.01. ADHD status in block 2 did not produce a significant increase in variance explained (*R*^2^ change = .02, F change (189) = 2.07, *p* = 0.15).

## 4 Discussion

Previous research has shown that ADHD is associated with measures of MW-S, and that both ADHD and MW-S are associated with similar cognitive and neural measures. This led to the hypothesis that the same cognitive-EEG correlates showing differences between individuals with ADHD and controls, and associated with MW-S in population samples, will also be associated with MW-S in individuals with ADHD [5]. Yet, to our knowledge, the present study is the first one to investigate the association between cognitive-EEG measures and MW-S in individuals with and without ADHD. Adults with ADHD showed EEG impairments in attention resource allocation to targets and non-targets (reduced P3 to Go and No-Go stimuli), attention selection (lower alpha suppression) and motor response activity (lower beta suppression) during response inhibition (No-Go trials), as well as response execution (reduced theta activation in the 500−1000 ms window). Individuals with ADHD further showed increased RTV, CE and OE. Higher self-reported MW-S was continuously associated with the same cognitive-EEG impairments (except number of errors) across the entire sample, and additionally with greater MRT and reduced attention allocation (theta ERSP) during processing of Go and No-Go stimuli (between 0 and 500 ms). When analysed together in the hierarchical regression analyses, ADHD diagnosis and MW-S did not independently account for any of the cognitive-EEG impairments, except for lower beta suppression. These findings (despite not providing direct evidence) may be consistent with the view [5] that these measures represent shared impairments associated with both ADHD and increased MW-S. Taken together, these results extend our understanding of the cognitive and neural processes associated with increased MW-S in individuals with ADHD, and suggest a common neurobiological basis underlying MW-S and the disorder.

Our analyses comparing adults with and without ADHD (aim 1) on EEG measures of attentional processes primarily focused on event-related modulations in brain oscillations derived through time-frequency EEG analyses, which provide fine-grained information on brain functioning [10]. In line with our hypotheses based on previous oscillatory findings in ADHD [37,49], individuals with ADHD showed reduced alpha and beta suppression over parietal regions during No-Go trials. These atypical patterns in alpha and beta suppression emerged as significant during the later time window (600−1000 ms), indicating that adults with ADHD are impaired during the interval between stimuli. In both groups and across time windows, alpha and beta suppressions were stronger during response inhibition (No-Go trials) than during response execution (Go trials), suggesting that the more cognitively demanding No-Go condition requires stronger attention selection and motor response activity, in line with findings from a population-based sample [59]. The emergence of differences between individuals with ADHD and controls in alpha and beta suppression only during the No-Go condition therefore suggests that during this task individuals with ADHD show a suboptimal ability to suppress alpha and beta activity during response inhibition, but intact suppression during response execution. Adults with ADHD further showed reduced evoked theta power compared to controls during Go trials, confirming our predictions based on previous literature [40,55]. Both ADHD and control groups showed higher theta power in the earlier stimulus-processing time window (0−500 ms) during No-Go trials than during Go trials, suggesting that the No-Go condition requires greater attention allocation after stimulus presentation. Since the ADHD group showed lower theta power compared to controls during Go trials, which was statistically significant in a later time window (500−1000 ms) but did not survive multiple-testing correction in the earlier window, our findings indicate reduced EEG power modulation during response execution to stimuli requiring a response. These findings are consistent with the few available studies on EEG brain-oscillatory markers in ADHD samples [39,34,37,55,60].

Impairments in time-frequency EEG measures in adults with ADHD were accompanied by an attenuated P3 amplitude to Go and No-Go stimuli, in line with previous findings in adult ADHD samples using the SART and other similar attentional tasks [19,35,[61], [62], [63]]. During the SART, an attenuated P3 in individuals with ADHD compared to controls, which emerged in trials requiring either response execution (Go condition) or response inhibition (in the No-Go condition), likely reflects an overall attentional resource deficit. Additionally, increased RTV and poor accuracy (number of errors) were also sensitive to ADHD-control differences, as expected from numerous previous studies of ADHD (Johnson et al., 2007; [18]. Together, our analyses comparing adults with and without ADHD extend previous studies of adult ADHD and converge in indicating impairments in several cognitive and brain functions related to attentional and inhibitory processes in adults with ADHD.

Our second analysis testing aim 2 further showed that the same indices of alpha suppression, beta suppression, theta power in the later window and P3, as well as of RTV, that were impaired in adults with ADHD were also continuously associated with self-reported MW-S. Since reduced alpha suppression is a marker of attention selection (i.e. the ability to filter out task-irrelevant information) [31], this EEG marker may represent a neural correlate underlying spontaneous task-unrelated thoughts, here potentially indexed by severity of MW-S. The associations of MW-S with parietal beta suppression during No-Go stimuli and with evoked theta oscillations following Go stimuli further suggest that increased MW-S is associated with a lower ability to inhibit motor responses and allocate attention to stimuli requiring a response, respectively. Reduced P3 was also associated with higher MW-S in this sample of individuals with and without ADHD, in line with previous studies in the general population [14]. We further found an association between increased MW-S and decreased early theta during both conditions, which was not significantly impaired in ADHD in this sample. This finding is consistent with a previous study linking increased early theta power with on-task episodes and reduced early theta power during off-task episodes using the SART [47]. At the cognitive-performance level, we found a novel association between MW-S and RTV, which was not investigated in previous studies. Increased MW-S was also related to slower responses, replicating previous work on MW [14]. The association with both omission and commission errors did not reach statistical significance. Since increased RTV in ADHD samples is thought to index attentional lapses and is linked with hypo-arousal and neural markers of attention allocation [22,62], this finding may suggest that MW-S is more related to impairments in attentional processes (e.g. RTV) than with inhibitory impairments (commission errors) that also characterise individuals with ADHD. This suggestion is further supported by the significant association between neural markers of attentional processes (i.e., alpha suppression) and behavioural markers of attentional processes (i.e., RTV), but a lack of an association of these neural measures with behavioural markers of inhibition (i.e., commission errors).

Finally, our analysis testing aim 3 involved hierarchical regressions to examine whether the cognitive-EEG measures sensitive to ADHD-control differences and associated with MW-S represented shared processes, potentially underlying both ADHD and MW-S, or whether MW-S has a unique relationship with these measures beyond ADHD or vice versa. The results indicate that MW did not have a unique contribution to most of the examined cognitive-EEG measures (RTV, No-Go and Go P3, No-Go alpha suppression, and Go theta power) beyond ADHD diagnosis, nor did ADHD beyond MW-S. Therefore, RTV, (No-Go/Go P3, Go theta and No-Go alpha appear to be shared correlates of both ADHD and MW-S. The exception was for No-Go parietal beta suppression, where MW-S explained a significant proportion of the variance beyond ADHD, while ADHD did not after MW-S was already included in the model. Potentially, the unique association between MW-S and No-Go parietal beta suppression might indicate that increased MW-S has a unique association with impairments associated with motor response activity beyond ADHD diagnosis itself. These results should be considered along with the high correlations that we reported between self-reported MW-S and ADHD symptoms in this sample [3]. Together, these findings are consistent with the view that there is a substantial degree of overlap in the cognitive-EEG underpinnings of clinical measures of ADHD and MW-S, in line with our hypothesis on the role of MW-S in ADHD [5].

The following limitations should be considered. First, the version of the SART used in this study may not be optimal for eliciting MW episodes, as it was relatively short (three 5-min blocks) and highly engaging due to fast presentation rate and high rate of targets compared to slower and unpredictable stimuli presentation and longer task duration (30−45 min) in previous mind wandering studies [47,14]. The fast presentation rate may also explain the lack of differences between individuals with ADHD and controls in ITC, previously reported in studies with longer inter-stimulus intervals [37,53,55,40], as fast presentation rate is associated with phasic consistency in the neural response across trials [64]. Of note, estimation of phase could be affected by task-induced changes in the power of oscillations or concurrent evoked responses [58]. However, since there were no differences between individuals with and without ADHD in theta ERSP in the early window (0−500 ms) where theta ITC was measured, it is unlikely that this occurred in the current study. Further, given the short inter-stimulus interval, the pre-stimulus period (−500 ms) used for EEG analyses was temporally close, though not overlapping, with the response to the preceding trial (occurring at 200−300 ms). Future work could therefore use a SART with a slower presentation rate and longer duration, potentially at least 30 min.

Future work could therefore use a SART with a slower presentation rate and longer duration, potentially at least 30 min. Second, due the lack of a direct measure of MW-S, we were also not able to explore neural impairments during episodes of MW-S. Such an approach has been adopted in previous brain oscillatory work on MW in the general population, which reported higher theta and lower alpha and beta activations around 300−400 ms after a MW episode compared to episodes of task focus [28,29]. This difference in methodology between previous ADHD and MW-S studies likely explains why we found associations of theta, alpha and beta power with our measure of MW-S severity and ADHD mostly in later time windows, from 500/600 ms onwards. Future studies should integrate MW probes during attentional tasks to measure neural activity during MW periods in individuals with and without ADHD as well as test the specificity of the association between these cognitive-EEG measures and MW-S by also including their association with MW-D. Third, MW-S has also been reported in other psychiatric disorders, not just in ADHD. The inclusion only of a control group without a history of mental illness did not allow us to examine whether the relationship between the cognitive-EEG measures and MW-S is specific to ADHD or could also characterise other forms of psychopathology. Future research should clarify this matter by comparing individuals with different psychiatric disorders. Fourth, although this study is one of the largest EEG investigations on adult ADHD to date and we detected medium-to-large effects as significant with the current sample size, the sample is still relatively small. Future studies using larger samples will be required to replicate these findings and detect potentially subtler effects.

In conclusion, these findings provide the first evidence for a close relationship between increased MW-S and neural markers of attention allocation, attention selection, motor response activity and response execution/inhibition that are impaired in individuals with ADHD. Our findings further suggest that that self-reported MW-S and ADHD diagnosis might largely be linked to the same electrophysiological processes. Future studies should focus on investigating these neural indices sensitive to both ADHD and MW-S in individuals with and without ADHD during paradigms explicitly designed to measure MW-S and applying causal modelling to provide more direct evidence for this relationship as well as in relation to MW-S assessed with different types of assessments, such as cognitive, online and experience-sampling measures.

## Funding

The OCEAN study was funded by Vifor Pharma (PADWUDB), awarded to Philip Asherson with King’s College London as sponsor. His research is supported by the NIHR Biomedical Research Centre at South London and Maudsley NHS Foundation Trust and King’s College London, NIHR/BRC (14/23/17) and an NIHR Senior Investigator award (NF-SI-0616-10040)

Natali Bozhilova’s research is supported by a studentship awarded by the 10.13039/501100007155Medical Research Council, as part of a doctoral training programme (DTP).

Dr Giorgia Michelini was in receipt of a fellowship funded by the National Institute for Health Research (NIHR) Biomedical Research Centre at South London and Maudsley NHS Foundation Trust and King’s College London.

The views expressed are those of the authors and not necessarily those of the NHS, the NIHR or the Department of Health and Social Care.

## Declaration of Competing Interest

Professor Jonna Kuntsi has given talks at educational events sponsored by Medice: all funds are received by King’s College London and used for studies of ADHD. Professor Philip Asherson has received honoraria for consultancy to Shire/Takeda, Flynn-Pharma, Eli-Lilly, Janssen, Novartis, Lundbeck and Medice; educational/research awards from Janssen, Shire, Lilly, Novartis, Flynn Pharma, Vifor Pharma, GW Pharma and QbTech; speaker at sponsored events for Shire/Takeda, Lilly, Novartis, Medice, Janssen-Cilag and Flynn Pharma
