# Supplementary material for: Electrophysiological correlates of spontaneous mind wandering in attention-deficit/hyperactivity disorder
Source: Behav Brain Res. 2020 Aug 5;391:112632. doi: 10.1016/j.bbr.2020.112632 (PMC7303944; doi:10.1016/j.bbr.2020.112632)
Supplement: Supplementary file 1 [file mmc1.docx]

**Supplementary material**

**Supplementary statistical analyses**

Due to a potential confounding effect of comorbid depressive/anxiety symptomatology, we performed comparisons between individuals with ADHD who were taking medication for depression/anxiety and individuals with ADHD who were not taking medication for depression/anxiety sensitivity (Supplementary Analysis 1).

Previous work (Arnau et al., 2019) suggests that inconsistency between brain oscillatory studies in the MW literature might be due to a differing choice of time window (e.g., splitting the window into an early and late window, or using 1s window). We therefore performed an additional analysis of ERSPs using a single time window of 1s (Supplementary Analyses 3).

Since previous work suggests that group differences in ITC indices might be driven by differences in power (van Diepen, & Mazaheri, 2018), we repeated the statistical analyses on theta ITC analyses controlling for theta ERSP in the early window (Supplementary Analysis 2).

To understand whether attenuation in neural process also relate to poorer cognitive performance, we performed correlations between cognitive performance and cognitive-EGG variables sensitive to case-control differences (Supplementary Analyses 4).

Since individuals with ADHD shower higher levels of MW than controls in this study, as expected (Bozhilova et al., 2018), we repeated Analyses 2 (investigating the association between MEWS and cognitive-EEG measures) with an additional interaction between group and the MEWS to check whether the association between the cognitive-EEG measures and the MEWS varies as a function of group (Supplementary Analyses 5).

**Supplementary Analysis 1: Differences between individuals with ADHD who are not taking medication for depression/anxiety and controls**

In line with Analysis 1, individuals with ADHD showed attenuation in most cognitive-EEG measures compared to controls (Supplementary Table 1), indicating that participants taking medication for anxiety or depression did not explain the difference between the ADHD and control groups.

**Supplementary Analysis 2: Differences between ADHD and control groups in ERSP variables over 1s time window**

*Alpha ERSP.* A significant main effect of condition (p<0.001), group (p=0.008) and a group-by-condition-by-time interaction (p=0.004) emerged for alpha ERSP. Post-hoc analyses revealed that compared to controls, Individuals with ADHD lower alpha suppression in the No-Go (p=0.004), but there were no group differences in the Go condition (p=0.07). Alpha suppression was lower in the Go compared to the No-Go condition (p<0.0001).

*Beta ERSP.* A significant main effect of condition (p<0.0001), but there was no main effect of group (p=0.63) or condition-by-group interaction (p=0.63) over central regions.

Over parietal regions, a significant main effect of condition (p<0.0001) and a significant condition-by-group interaction (p=0.01) emerged, but there was no main effect of group (p=0.06). Post-hoc analyses revealed that beta suppression over parietal regions was lower in the ADHD group compared to controls during the No-Go condition (p=0.02), but there were no group differences in the Go condition (p=0.43). Beta suppression was lower in the Go compared to the No-Go condition (p<0.0001).

*Theta ERSP.* A significant main effect of condition (p<0.001) was observed for theta ERSP, but there was no main effect of group (p=0.23) or a group-by-condition-by-time interaction (p=0.38).

**Supplementary Analyses 3: Differences between individuals with ADHD and controls in theta ITC after controlling for theta ERSP (0-500ms)**

When controlling for theta ERSP in the early window (0-500ms), the main effect of condition (p<0.0001), group (p=0.06) and group-by-condition (p=0.11) remained the same as analysis 1 for theta ITC.

**Supplementary Analysis 4: Correlations between cognitive performance and EEG measures sensitive to group differences**

ERP variables (P3 Go, P3 No-Go) were negatively and significantly correlated with all cognitive performance measures (CE, OE and RTV), indicating lower P3 amplitude with greater error rate and increased RTV. Whereas ERSP variables (Alpha ERSP No-Go and Beta ERSP No-Go) in the later window were only correlated with RTV, indexing lower alpha and beta suppression with increased RTV. There was no link between theta ERSP Go (500-1000ms) and cognitive performance measures (Supplementary Table 2).

**Supplementary Analysis 5: Associations between cognitive-EEG measures and the MEWS as a function of group**

The association between MRT, theta ERSP Go (500-1000 ms) and the MEWS disappeared after adding group to the model. None of the other associations between cognitive-EEG measures and the MEWS changed or varied as a function of group (Supplementary Table 3).

**Supplementary Table 1. Differences in cognitive-EEG measures between individuals with ADHD who are not taking medication for depression/anxiety and controls**

|  | | **ADHD** | **Control** |  |  |
| --- | --- | --- | --- | --- | --- |
|  | | **Mean ± SD** | **Mean ± SD** | **d** | **p** |
| OE | | 7.16 ± 8.50 | 2.79 ± 4.64 | *0.64* | .003* |
| CE | | 10.54 ± 4.71 | 7.20 ± 4.20 | *0.75* | .003* |
| MRT (ms) | | 323.11±56.49 | 308.99±43.76 | 0.28 | .22 |
| RTV (ms) | | 125.50± 62.71 | 83.37± 26.99 | **0.87** | .001** |
| P3 | Go | 1.01 ± 0.64 | 1.33 ± 0.68 | 0.48 | .040* |
|  | No-Go | 2.64 ± 1.29 | 3.83 ± 1.58 | **0.83** | .001* |
| Alpha ERSP | Go (200-600 ms) | -1.80 ± 1.42 | -2.49 ± 1.52 | 0.47 | .046* |
|  | No-Go (200-600 ms) | -2.83 ± 2.17 | -3.93 ± 2.28 | 0.49 | .036* |
|  | Go (600-1000 ms) | 0.20 ± 0.44 | -0.14 ± 0.56 | 0.12 | .176 |
|  | No-Go (600-1000 ms) | -1.75 ± 1.40 | -3.21 ± 1.93 | **0.87** | <0.001** |
| Beta ERSP (Central) | Go (200-600ms) | -1.58 ± 0.77 | -1.56 ± 0.72 | 0.03 | .562 |
|  | No-Go (200-600 ms) | -1.94 ± 1.14 | -1.94 ± 1.08 | 0.01 | .998 |
|  | Go (600-1000 ms) | 0.15 ± 0.23 | 0.16 ± 0.20 | 0.05 | .562 |
|  | No-Go (600-1000 ms) | -0.81 ± 0.88 | -1.06 ± 0.75 | 0.31 | .218 |
| Beta ERSP (Parietal) | Go (200-600 ms) | 1.56 ± 0.70 | -1.43 ± 0.70 | 0.19 | .448 |
|  | No-Go (200-600 ms) | -2.38 ± 1.02 | -1.88 ± 1.24 | 0.44 | .112 |
|  | Go (600-1000 ms) | 0.16 ± 0.28 | 0.16 ± 0.13 | 0.01 | .779 |
|  | No-Go (600-1000 ms) | -0.94 ± 0.80 | -0.54 ± 0.48 | *0.61* | .040* |
| Theta ERSP | Go (0-500 ms) | 1.89 ± 0.87 | 1.34 ± 0.78 | *0.67* | .069 |
|  | No-Go (0-500 ms) | 4.17 ± 1.57 | 3.76 ± 1.92 | 0.23 | .126 |
|  | Go (500-1000 ms) | 0.43 ± 0.40 | 0.20 ± 0.21 | *0.72* | .012* |
|  | No-Go (500-1000 ms) | 0.13±1.07 | 0.45 ± 1.01 | 0.31 | .364 |
| Theta ITC | Go (0-500 ms) | 0.33 ± 0.11 | 0.31 ± 0.09 | 0.20 | .272 |
|  | No-Go (0-500 ms) | 0.50 ± 0.10 | 0.45 ± 0.13 | 0.43 | .585 |

Abbreviations: ADHD- Attention-Deficit/Hyperactivity Disorder, MRT- Mean Reaction Time, RTV- Reaction Time Variability, CE- commission errors, OE- omission errors, ERSP-Event-related spectral perturbations, ITC- inter-trial phase coherence

Notes: *significant at p≤0.05, **p≤0.001 Bold: d≥.80 indicating large effect size, Italics: d≥.50 indicating a medium effect size, d≥.20 indicating a small effect size

**Supplementary Table 2.** Correlations between cognitive performance and EEG variables sensitive to group differences

|  |  | **CE** | **OE** | **RTV** |
| --- | --- | --- | --- | --- |
| **P3** | Go | -.23* | -.25* | -.39** |
|  | No-Go | -.21* | -.25* | -.33** |
| **Alpha ERSP** | No-Go (600-1000ms | .15 | .16 | .21* |
| **Beta parietal ERSP** | No-Go (600 -1000ms) | .12 | .13 | .20* |
| **Theta ERSP** | Go (500-1000 ms) | -.16 | -.19 | -.17 |

RTV- Reaction Time Variability, CE- commission errors, OE- omission errors,

ERSP-Event- related spectral perturbations, ITC- inter-trial phase coherence

Notes: *significant at p≤0.05, **significant at p≤0.001

**Supplementary Table 3. Association between cognitive-EEG measures and the MEWS as a function of group**

|  | | **MEWS** | | | **MEWS x group** | | |
| --- | --- | --- | --- | --- | --- | --- | --- |
|  | | **p** | | | **p** | | |
| **OE** | | 0.02 | | | 0.85 | | |
| **CE** | | 0.08 | | | 0.85 | | |
| **MRT (ms)** | | 0.06 | | | 0.14 | | |
| **RTV (ms)** | | 0.005* | | | 0.72 | | |
| **P3** | Go | | | 0.01* | | 0.68 |  |
|  | No-Go | | | 0.0002** | | 0.13 |  |
| **Alpha ERSP** | Go (200-600 ms) | | | 0.21 | | 0.21 |  |
|  | No-Go (200-600 ms) | | | 0.13 | | 0.37 |  |
|  | Go (600-1000 ms) | | | 0.44 | | 0.29 |  |
|  | No-Go (600-1000 ms) | | | 0.001** | | 0.33 |  |
| **Beta ERSP (Central)** | Go (200-600ms) | | | 0.81 | | 0.67 |  |
|  | No-Go (200-600 ms) | | | 0.81 | | 0.46 |  |
|  | Go (600-1000 ms) | | | 0.27 | | 0.37 |  |
|  | No-Go (600-1000 ms) | | | 0.11 | | 0.10 |  |
| **Beta ERSP (Parietal)** | Go (200-600 ms) | | | 0.64 | | 0.51 |  |
|  | No-Go (200-600 ms) | | | 0.35 | | 0.31 |  |
|  | Go (600-1000 ms) | | | 0.96 | | 0.23 |  |
|  | No-Go (600-1000 ms) | | | 0.01* | | 0.63 |  |
| **Theta ERSP** | Go (0-500 ms) | | | 0.004* | | 0.08 |  |
|  | No-Go (0-500 ms) | | | 0.009* | | 0.15 |  |
|  | Go (500-1000 ms) | | | 0.03 | | 0.17 |  |
|  | No-Go (500-1000 ms) | | | 0.02 | | 0.72 |  |
| **Theta ITC** | Go (0-500 ms) | | 0.08 | | | 0.12 |  |
|  | No-Go (0-500 ms) | | 0.05 | | | 0.89 |  |

RTV- Reaction Time Variability, CE- commission errors, OE- omission errors, ERSP-Event-related spectral perturbations, ITC- inter-trial phase coherence, MEWS- Mind wandering excessively scale

Notes: *significant at p≤0.05 **significant at p≤0.001
